# Identification and Quantification of Flavonoids and Phenolic Acids in Burr Parsley (*Caucalis platycarpos* L.), Using High-Performance Liquid Chromatography with Diode Array Detection and Electrospray Ionization Mass Spectrometry

**DOI:** 10.3390/molecules14072466

**Published:** 2009-07-09

**Authors:** Ana Plazonić, Franz Bucar, Željan Maleš, Ana Mornar, Biljana Nigović, Nikola Kujundžić

**Affiliations:** 1 Agency for Medicinal Products and Medical Devices, Ksaverska cesta 4, 10000 Zagreb, Croatia; 2 Institute of Pharmaceutical Sciences, University of Graz, A-8010, Austria; 3 Faculty of Pharmacy and Biochemistry, University of Zagreb, Ante Kovačića 1, 10000 Zagreb, Croatia

**Keywords:** *Caucalis platycarpos* L., flavonoids, phenolic acids, RP-HPLC-DAD-MS/MS

## Abstract

A sensitive method coupling high-performance liquid chromatography (HPLC) with diode-array detector (DAD) and electrospray ionization mass spectrometry (MS) was optimized for the separation and identification of phenolic acids, flavonoid glycosides and flavonoid aglycones in the extract of burr parsley (*Caucalis platycarpos* L.). Fragmentation behavior of flavonoid glycosides and phenolic acids were investigated using ion trap mass spectrometry in negative electrospray ionization. The MS, MS^n^ and UV data together with HPLC retention time (*T_R_*) of phenolic acids and flavonoids allowed structural characterization of these compounds. Caffeoylquinic acid (CQA) isomers, *p*-coumaroyl-quinic acids (*p*-CoQA), feruloylquinic acids (FQA), dicaffeoylquinic acids (diCQA), luteolin-7-*O*-rutinoside, apigenin-7-*O*-rutinoside as well as isolated chrysoeriol-7-*O*-rutinoside have been identified as constituents of *C. platycarpos* for the first time. An accurate, precise and sensitive LC-DAD method for quantification of four phenolic acids (3-*O*-caffeoylquinic, caffeic, *p*-coumaric, *o*-coumaric acid), four flavonoid glycosides (luteolin-7-*O*-glucoside, apigenin-7-*O*-glucoside, quercetin-3-*O*-galactoside, quercetin-3-*O*-rhamnoside), and three flavonoid aglycones (luteolin, apigenin, chrysoeriol) in *C.* *platycarpos* extract was validated in terms of linearity, limit of detection, limit of quantification, precision and accuracy. 3-*O*-caffeoylquinic acid was the predominant phenolic acid and luteolin-7-*O*-glucoside was the predominant flavonoid glycoside.

## 1. Introduction

Due to their high frequency and mortality malignant diseases are currently one of the main issues in medicine. A lot of attention is given to natural compounds because they can interfere with tumor growth. Burr parsley (*Caucalis platycarpos* L., *Caucalis daucoides* L.), an annual plant growing on clayish lime-containing soils in the Mediterranean and Central Europe, has been used in folk medicine for the treatment of certain types of tumor. Water extracts of the aboveground parts of this plant showed a remarkable antitumor activity in rats and mice. It is probable that antitumor activity is a consequence of stimulation of the immunological system of the host. *C*. *platycarpos* water extract showed activation of T-lymphocytes and NK cells and stimulated the spleen as a lymphatic organ, to produce antitumor factors [1]. However, chemical composition is only partially defined from general phytochemical and chromatographic investigations. Phenolic compounds, including phenolic acids and flavonoids, and polysaccharides are considered to be the major bioactive compounds in *C. platycarpos* [2].

Flavonoids are one of the most important groups of bioactive compounds in plants, which exist in the free aglycones and the glycoside forms showing a diverse structure and a broad range of biological activities. Flavonoids include several classes of compounds with similar structure having a C6-C3-C6 flavone skeleton. They are differentiated on the degree of unsaturation and oxidation of the three carbon segment. Within different subclasses further differentiation is based on the number and nature of substituent groups attached on the rings. Mostly they occur in *O*-glycosidic forms with a number of sugars such as glucose, galactose, rhamnose, arabinose, xylose and rutinose but they are also present as *C*-glycosides. Flavonoid glycosides have many isomers with the same molecular weight but different aglycone and sugar component at different positions attaching on the aglycone ring [3,4].

Naturally occurring phenolic acids are phenylpropanoids with an aromatic ring and attached three carbon side chain. Caffeic, ferulic and *p*-coumaric acid, as hydroxycinnamic acids, are almost ubiquitous. Phenolic acids are distributed in nature in their free and bound forms, as esters and glycosides. Chlorogenic acids are a family of esters formed between *trans* cinnamic acids and (-)-quinic acid (1L-1(OH),3,4/5-tetrahydroxycyclohexanecarboxylic acid). A subgroup of chlorogenic acid is defined by the number and identity of the constituent cinnamic acids, and there are usually several isomers within each subgroup. Many plants produce chlorogenic acids in which esterification occurs at positions 3, 4 and 5 of the quinic acid moiety. Esterification at position 1 is less frequent, but 1-acyl chlorogenic acids are found in some *Asteraceae* [5,6,7].

Flavonoids and phenolic acids have protective role in carcinogenesis, inflammation, atherosclerosis, thrombosis and have high antioxidant capacity. Furthermore, flavonoids have been reported as aldose reductase inhibitors blocking the sorbitol pathway that is linked to many problems associated with diabetes [8,9,10,11,12]. Flavonoids interact with various enzymatic systems. Their inhibition of the enzymes cyclooxygenase and lipooxygenase results in a decrease of platelet activation and aggregation, protection against cardiovascular diseases, cancer chemoprevention and their anti-inflammatory activity [13,14,15,16,17]. Many other biological activities are attributed to flavonoids and phenolic acids: antiviral, antimicrobial, antihepatotoxic, antiosteoporotic, antiulcer, immunomodulatory, anti-proliferative and apoptotic activity [18,19,20,21,22,23,24,25,26].

The purpose of this research was to identify phenolic compounds, quantify main flavonoids and phenolic acids and to isolate a flavonoid glycoside that was found to be characteristic for *C. platycarpos*. A sensitive, accurate and specific method coupling high performance liquid chromatography (HPLC) with diode array detector (DAD) and electrospray ionization mass spectrometry (MS) was developed for the separation and identification of phenolic acids, flavonoid glycosides and aglycones in the methanolic extract of *C. platycarpos*.

The molecular masses of phenolic acids and flavonoids were assigned by electrospray ionization mass spectrometry. The subsequent structure characterization was carried out by a tandem mass spectrometric method. Fragmentation behavior of flavonoid glycosides and phenolic acids was investigated using ion trap mass spectrometry in negative mode. The fragmentation rule in mass spectrum offers the ability to identify the related unknown compounds. The MS, MS^n^ and UV data together with HPLC retention time (*T_R_*) of phenolic acids and flavonoids allowed structural characterization of these compounds. Isolated chrysoeriol-7-rutinoside was analyzed after elution and by direct injection into the MS system (MS^n^, n up to 3), by ^1^H-NMR, ^13^C-NMR, IR and UV/VIS (with "shift reagents").

## 2. Results and Discussion

### 2.1. Optimization of chromatographic conditions

A method coupling high-performance liquid chromatography (HPLC) with diode-array detector (DAD) and electrospray ionization mass spectrometry with an ion trap analyser was optimized for the separation and identification of phenolic acids, flavonoid glycosides and flavonoid aglycones in the extract of *C. platycarpos*. Different mobile phase compositions were screened to obtain chromatograms with good resolution within an acceptable time of analysis. Ten mM ammonium formate in water-acetonitrile 95:5 (v/v), pH 4.0 adjusted with formic acid, as solvent A, and 10 mM ammonium formate in water-acetonitrile 50:50 (v/v) pH 4.0 adjusted with formic acid, as solvent B, were chosen for the gradient elution. Changes in the pH value of the mobile phase had a significant effect on the resolution of compounds, especially the phenolic acids. Formic acid, acetic acid, trifluoroacetic acid, ammonium acetate and ammonium formate are volatile and thus compatible with LC/MS system. Because acetic acid was found to have weak ion-pairing capacity, ammonium formate (10 mM) was used to buffer the mobile phase at pH 4.0. The higher concentration of acid in mobile phase (lower pH values) ensures better sample separation but shortens the HPLC column lifetime and affects ESI ionization.

**Figure 1:**
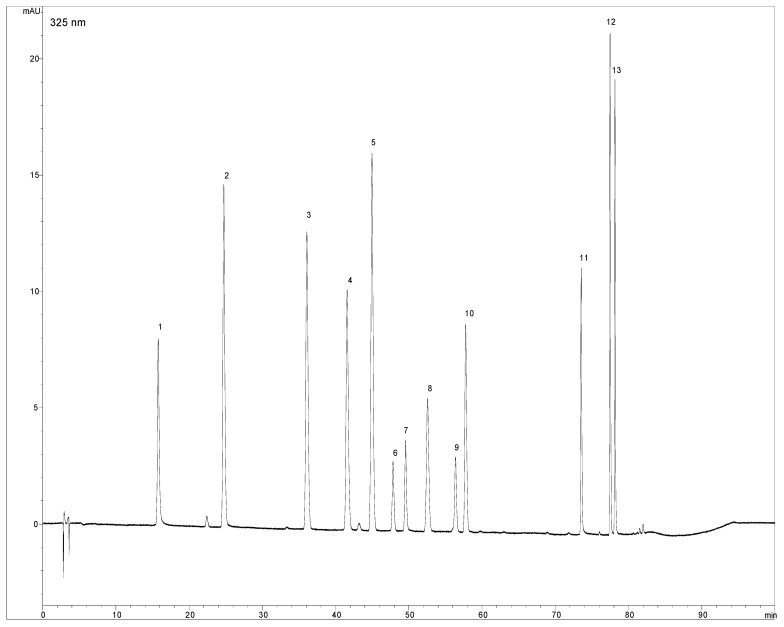
HPLC chromatogram of standard solution mixture (0.008 mg/mL) at 325 nm.

275 nm, 310 nm, 325 nm and 350 nm were chosen as monitoring wavelengths according to absorption maxima of analytes. Apigenin and chrysoeriol were found to be eluted closely together, but the differences in their absorption spectra were utilized for their quantification, by comparing their chromatograms recorded at 325 nm, for apigenin, and 350 nm, for chrysoeriol. The HPLC-DAD chromatogram of the standard solution mixture at 325 nm is shown in Figure 1.

1. 3-*O*-caffeoylquinic acid; 2. caffeic acid; 3. *p*-coumaric acid; 4. ferulic acid; 5. isoferulic acid; 6. quercetin-3-*O*-galactoside; 7. luteolin-7-*O*-glucoside; 8. *o*-coumaric acid; 9. quercetin-3-*O*-rhamnoside; 10. apigenin-7-*O*-glucoside; 11. luteolin; 12. apigenin; 13. Chrysoeriol.

For MS analysis the negative ion mode of ESI was selected, because it provided extensive structure information for most flavonoids and phenolic acids present in *C. platycarpos*. In addition to detection of the deprotonated molecular ions, collision induced dissociation (CID) was performed in the MS^2^ and MS^3^, and the resulting product ions were used as fingerprints of each component.

### 2.2. Validation data

The optimized RP-HPLC-DAD method was validated for the simultaneous analysis of six phenolic acids (3-*O*-caffeoylquinic acid, caffeic acid, ferulic acid, isoferulic acid, *p*-coumaric acid, o-coumaric acid), four flavonoid glycosides (luteolin-7-*O*-glucoside, quercetin-3-*O*-galactoside, quercetin-3-*O*-rhamnoside, apigenin-7-*O*-glucoside) and three flavonoid aglycones (luteolin, apigenin and chrysoeriol) in terms of linearity, limit of detection, limit of quantification, precision and accuracy.

The calibration curves were obtained by the external standard method on six levels of concentration of standard mixtures, with three injections per level. Chromatogram peak areas on 275 nm for *o*-coumaric acid; 310 nm for *p*-coumaric acid; 325 nm for 3-*O*-caffeoylquinic acid, caffeic acid, ferulic acid, isoferulic acid, apigenin-7-*O*-glucoside and apigenin; 350 nm for quercetin-3-*O*-galactoside, luteolin-7-*O*-glucoside, quercetin-3-O-rhamnoside, luteolin and chrysoeriol were plotted against the known concentrations of the standard solutions to establish the calibration equations. A linear regression equation was calculated by the least squares method. The detection limit (LOD) and limit of quantification (LOQ) were calculated from the residual standard deviation of the regression (σ) line and the slope (S) as follows: LOD = 3.3σ/S; LOQ = 10σ/S.

Three different concentrations of standard mixtures (0.005; 0.015 and 0.030 mg/mL) were used for intra- and interday precision testing. The areas under curves and retention times of the three consecutive injections, performed at each concentration on three different days, were used to calculate % RSD (relative standard deviation) interday precision. Intraday precision data for peak areas and retention times were calculated from six non-consecutive injections, performed at each concentration on the same day. The linear range, regression equation and correlation coefficient of each analytes, LOD and LOQ values, interday and intraday precision are summarized in Table 1.

HPLC accuracy was determined by recovery tests analyzing sample extracts spiked with three different standard mixture concentrations (0.005; 0.015; and 0.046 mg/mL). Recovery was expressed as the percent mean ratio of the measured added concentration to nominal value. Recovery data showing good accuracy are presented in Table 2. The sample solution kept at 4 °C was found to be stable for 48 h.

Peaks were assigned based on the retention time, UV spectra of the standard compounds using HPLC-DAD and the peak identities were further confirmed by HPLC-ESI-MS^n^ (n = up to 3). The eleven phenolic components were simultaneously determined by the proposed HPLC-UV method by means of external standard method. In some cases where the compounds were present in lower concentrations, the sample was concentrated to proper volume in order to match with the linear range. 3-*O*-caffeoylquinic acid was the predominant phenolic acid whereas luteolin-7-*O*-glucoside was the predominant flavonoid glycoside in *C. platycarpos* methanolic extract. Data of quantitative analyses are expressed as mean ± standard deviation and are listed in Table 3. Furthermore, methanolic extract of *C. platycarpos* was shown to contain phenolic acids in free forms and as phenolic acid esters. The content of the phenolic acid esters and some flavonoid glycosides was not determined because related standards are not commercially available. Their structure was elucidated by tandem MS.

### 2.3. MS analysis and identification

**Figure 2:**
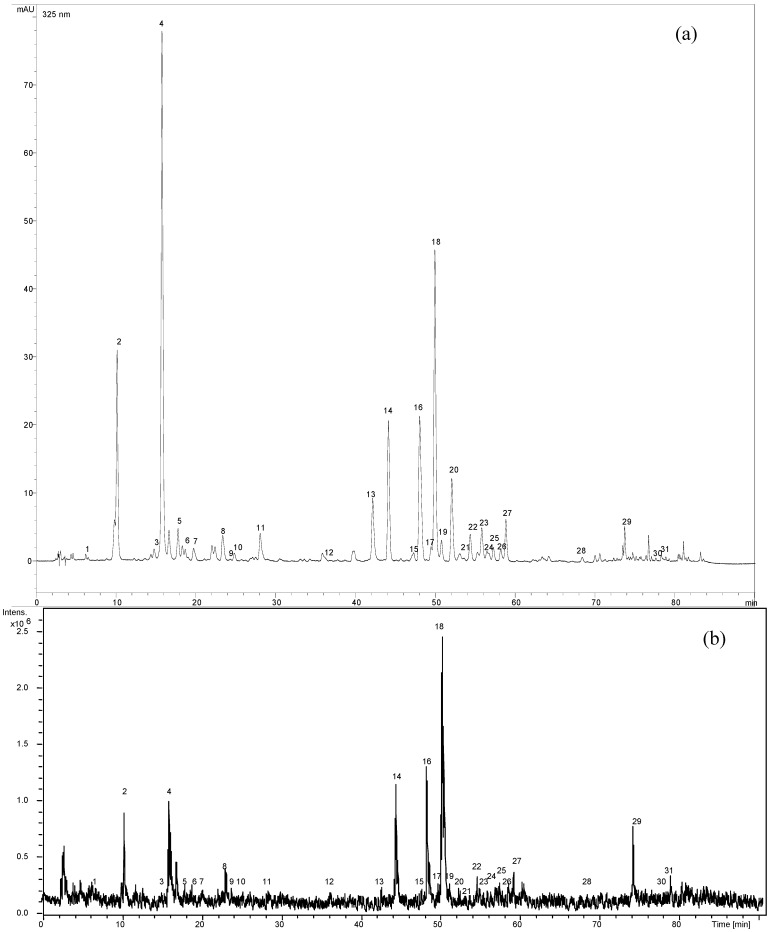
HPLC-DAD chromatogram of methanolic extract of *C. platycarpos*, λ=325 nm (a); TIC chromatogram of methanolic extract of *C. platycarpos* from HPLC-(-) ESI-MS (b). Peak identities are numbered in Table 4 and Table 5.

In this study, a total of 31 compounds were characterized. Eleven of them were unambiguously identified by comparing retention times (*T_R_*), UV and MS data with those of the reference standards. The possible structures of another 20 peaks in the chromatogram were tentatively characterized on the basis of literature data. The HPLC-DAD chromatograms and total ion chromatograms (TIC) in negative mode of the extracts of *C. platycarpos* are shown in Figure 2.

The application of MS to the analysis of flavonoid glycosides has increased with the development of so called, “soft” ionization techniques. Compounds of this class are polar, non volatile and thermally labile [27].

**Table 1:** Detection wavelength (λ), retention time (*T_R_*), linear regression and precision data.

**Compounds**	**λ** (nm)	***T_R_*** (min)	**Regression equation^1^**	**Linear** **(working) range** (mg/mL)	**R^2^**	**LOD** (mg/mL)	**LOQ** (mg/mL)	**Precision (RSD % *T_R_*, AUC^2^)**				
								**Conc.** (mg/mL)	**Intra-day**		**Inter-day**	
									*T_R_*	AUC	*T_R_*	AUC
3-*O*-caffeoylquinc acid	325	15.705 ± 0.197	y = 18.882x - 0.010	0.003-0.160 (0.005-0.100)	0.9999	0.001	0.002	0.005	0.20	0.57	0.48	1.09
								0.015	0.14	0.83	0.40	1.10
								0.030	0.29	0.69	1.01	0.52
Caffeic acid	325	24.770 ± 0.138	y = 29.500x + 0.027	0.004-0.140 (0.005-0.050)	0.9989	0.001	0.003	0.005	0.15	1.21	0.30	1.40
								0.015	0.07	1.15	0.29	1.04
								0.030	0.23	1.09	0.34	1.01
*p*-Coumaric acid	310	36.057 ± 0.135	y = 46.190x + 0.022	0.005-0.120 (0.005-0.080)	0.9997	0.001	0.005	0.005	0.10	1.19	0.22	1.35
								0.015	0.12	1.15	0.25	1.15
								0.030	0.17	1.24	0.20	1.08
Ferulic acid	325	41.721 ± 0.436	y = 26.745x + 0.016	0.004-0.120 (0.005-0.050)	0.9985	0.001	0.003	0.005	0.10	0.97	0.20	1.05
								0.015	0.08	1.12	0.25	1.11
								0.030	0.10	0.77	0.17	0.75
Isoferulic acid	325	45.075 ± 0.436	y = 40.266x + 0.012	0.004-0.100 (0.005-0.080)	0.9996	0.001	0.003	0.005	0.09	1.18	0.18	1.10
								0.015	0.11	1.00	0.26	0.89
								0.030	0.07	0.71	0.14	0.73
Quercetin-3-β-*O*-galactoside	350	47.960 ± 0.105	y = 7.903x + 0.006	0.004-0.120 (0.005-0.050)	0.9984	0.002	0.005	0.005	0.08	1.37	0.17	1.24
								0.015	0.13	1.18	0.27	1.05
								0.030	0.05	1.02	0.08	1.14
Luteolin-7-β-*O*-glucoside	350	49.733 ± 0.273	y = 12.011x - 0.005	0.004-0.200 (0.005-0.120)	0.9999	0.001	0.005	0.005	0.09	1.49	0.15	1.43
								0.015	0.11	1.15	0.25	1.10
								0.030	0.03	1.09	0.08	1.02
*o*-Coumaric acid	275	52.748 ± 0.402	y = 29.455x + 0.008	0.004-0.140 (0.005-0.050)	0.9998	0.001	0.002	0.005	0.14	1.35	0.15	1.27
								0.015	0.08	1.07	0.20	1.04
								0.030	0.11	0.95	0.10	0.91
Quercetin-3-*O*-α-rhamnoside	350	56.503 ± 0.102	y = 8.652x -0.001	0.005-0.120 (0.005-0.080)	0.9997	0.001	0.004	0.005	0.08	1.95	0.14	1.72
								0.015	0.09	1.24	0.22	1.21
								0.030	0.06	1.77	0.07	1.57
Apigenin-7-*O*-glucoside	325	57.864 ± 0.101	y = 16.860x + 0.016	0.004-0.100 (0.005-0.050)	0.9995	0.001	0.004	0.005	0.08	1.26	0.13	1.17
								0.015	0.06	1.14	0.20	1.02
								0.030	0.08	0.95	0,10	0.91
Luteolin	350	73.574 ± 0.051	y = 20.951x - 0.011	0.004-0.120 (0.004-0.050)	0.9999	0.001	0.003	0.005	0.03	1.32	0.03	1.36
								0.015	0.04	1.13	0.08	1.09
								0.030	0,01	0.90	0.03	0.85
Apigenin	325	77.496 ± 0.032	y = 22.516x - 0.001	0.002-0.120 (0.002-0.050)	0.9990	0.001	0.002	0.005	0.02	1.15	0.02	1.02
								0.015	0.04	1.08	0.06	1.02
								0.030	0.01	0.86	0.02	0.77
Chrysoeriol	350	78.155 ± 0.045	y = 25.205x + 0.025	0.005-0.120 (0.005-0.080)	0.9987	0.002	0.005	0.005	0.27	1.15	0.43	1.07
								0.015	0.04	1.16	0.06	1.08
								0.030	0.01	0.62	0.02	0.62

^1^ y = ax+b; where x is concentration in mg/mL, and y is area under curve at the selected wavelength. ^2^ AUC = area under curve.

**Table 2:** Recovery and accuracy data.

Compounds	Recovery		
	Amount added	Recovery	RSD
	(mg/mL)	(%)	(%)
3-*O*-caffeoylquinic acid	0.005	99.14	1.41
	0.015	99.81	1.17
	0.046	98.88	0.55
Caffeic acid	0.005	99.07	0.70
	0.015	99.02	0.77
	0.046	99.52	1.01
*p*-Coumaric acid	0.005	99.03	1.05
	0.015	99.64	0.50
	0.046	99.56	1.01
Ferulic acid	0.005	98.78	1.02
	0.015	99.76	1.14
	0.046	99.75	0.14
Isoferulic acid	0.005	99.79	1.89
	0.015	100.46	0.67
	0.046	99.24	0.15
Quercetin-3-*O*-β-galactoside	0.005	98.42	1.53
	0.015	100.75	0.58
	0.046	101.53	0.59
Luteolin-*7-O*-β-glucoside	0.005	101.11	1.67
	0.015	99.79	0.99
	0.046	100.33	0.83
*o*-Coumaric acid	0.005	99.79	1.81
	0.015	100.35	0.63
	0.046	99.26	0.19
Quercetin-3-*O*-α-rhamnoside	0.005	98.74	1.97
	0.015	98.52	0.93
	0.046	100.65	0.64
Apigenin-7-*O*-β-glucoside	0.005	98.62	1.27
	0.015	99.70	0.39
	0.046	99.53	0.71
Luteolin	0.005	99.06	1.62
	0.015	100.50	0.58
	0.046	100.38	0.67
Apigenin	0.005	100.58	1.82
	0.015	99.93	0.72
	0.046	100.05	0.40
Chrysoeriol	0.005	98.78	1.17
	0.015	100.88	0.18
	0.046	99.87	0.25

**Table 3:** Phenolic compounds in the aboveground parts of *C. platycarpos* (mg/kg dry matter).

Compound	Average amount ± SD
3-*O*-caffeoylquinic acid	1,023.25 ± 5.44
Caffeic acid	3.82 ± 0.16
*p*-Coumaric acid	0.72 ± 0.02
Ferulic acid	below LOQ
Isoferulic acid	below LOQ
Quercetin-3-*O*-β-galactoside	41.88 ± 0.86
Luteolin-*7-O*-β-glucoside	1,366.91 ± 7.50
*o*-Coumaric acid	2.42 ± 0.05
Quercetin-3-*O*-α-rhamnoside	37.07 ± 0.27
Apigenin-7-*O*-β-glucoside	15.30 ± 0.33
Luteolin	58.07 ± 0.09
Apigenin	5.011 ± 0.09
Chrysoeriol	3.19 ± 0.04

Values are means ± SD, n = 5.

### 2.4. Structure characterization of the flavonoids by MS^n^

Mass spectrometric methods can be used to obtain information on the carbohydrate sequence and the aglycone. Flavonoid aglycones are structurally diverse group of natural products. The most important variations in their structure are in the level of oxygenation (hydroxyl or methoxyl groups) and the point of attachment of ring B (flavonoids and isoflavonoids). When tandem mass spectrometric experiments are performed on instruments with ion trap analyzers, it is possible to perform tandem experiments many times (MS^n^) on sequential product ions. From the mass spectra of flavonoid glycosides using tandem MS we can obtain molecular mass, structure of the aglycone (pattern of hydroxylation on aglycone, point of attachment of ring B on ring C), information about acylation of sugar hydroxyl groups, possible methylation or sulphation of aglycone hydroxyl(s), number of sugar rings, their configuration and in some cases placement of glycosidic bonds. Negative ion mode was selected because previous results suggested that negative mode was more sensitive than positive mode. The [M-H]^-^ ions were selected for collision induced fragmentation (CID) to produce MS/MS spectra. The prominent MS/MS ions were then selected for further MS^3^ analysis.

The screening made us found the target compounds, and their structures were further elucidated using tandem MS. In the structure characterization, we first judged if the flavonoid glycoside is a C-glycosylated. The carbon-carbon bond of C-glycosyl flavonoids is resistant to rupture and in C-glycosides mainly the fragmentation of the sugar unit is observed. Fragmentation pathway of O-glycosylated flavonoids starts with the cleavage of the glycosydic bonds and elimination of the sugar moieties with charge retention on aglycone. In compounds containing two or more sugars to the same aglycone carbon, ions arising from the cleavage of the glycosidic bonds between sugar units are weak. Although the aglycone and the glycane were all identified, the accurate structure of the flavonoids glycoside could not be always determined because identity and the site of connection of monosaccharide cannot be determined by LC-MS. The structures of compounds were finally identified by comparison with literature [27,28,29,30,31,32,33].

**Figure 3:**
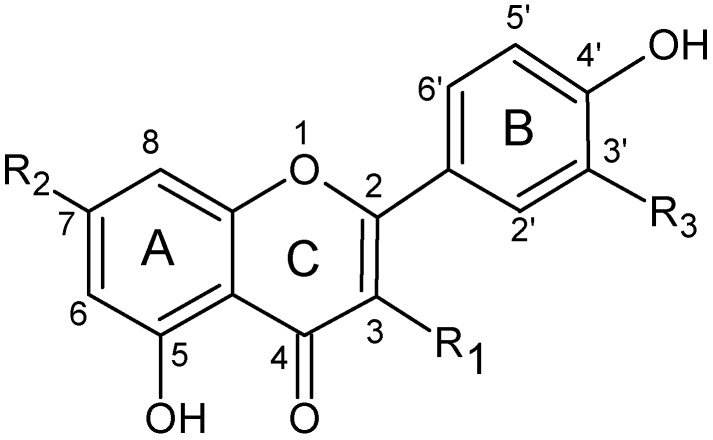
Chemical structure of flavonoids present in *C. platycarpos*.

The data of retention times (*T_R_*), molecular weight and the maximal UV wavelength (λ_max_) and MS^n^ data of the flavonoids detected in the extracts are listed in Table 4. The compounds were identified according to their fragmentation data and UV absorption and their structures are shown in Figure 3. 

**Table:** 

Flavonoid	R_1_	R_2_	R_3_
Apigenin	H	OH	H
Luteolin	H	OH	OH
Chrysoeriol	H	OH	OCH_3_
Apigenin-7-*O*-glucoside	H	7-*O*-β-D-glucopyranoside	H
Apigenin-7-*O*-rutinoside	H	7-*O*-[α-L-rhamnopyranosyl-β-D-glucopyranoside]	H
Luteolin-7-*O*-glucoside	H	7-*O*-β-D-glucopyranoside	OH
Luteolin-7-*O*-rutinoside	H	7-*O*-[α-L-rhamnopyranosyl-β-D-glucopyranoside]	OH
Chrysoeriol-7-*O*-rutinoside	H	7-*O*-[α-L-rhamnopyranosyl-β-D-glucopyranoside]	OCH_3_
Quercetin-3-*O*-galactoside	3-*O*-β-D-galactoside	OH	OH
Quercetin-3-*O*-rhamnoside	3-*O*-α-D-rhamnoside	OH	OH

Major diagnostic fragments of flavonoid aglycone identification are those involving the cleavage of two C-C bonds of the C-ring giving two fragment ions which provide information about the number and type of substituents in A- and B- rings. These fragment ions are designated according to the nomenclature proposed by Ma et al [36]. For free aglycone ^i,j^A and ^i,j^B labels refer to the fragments containing intact A- and B-rings, respectively, in which the superscripts i and j indicate the C-ring bonds that have been broken.

**Figure 4:**
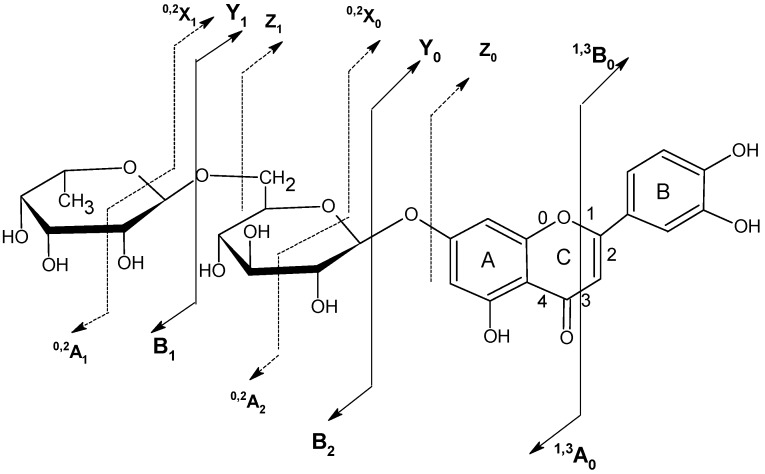
Ion nomenclature and major fragments illustrated on luteolin-7-*O*-rutinoside.

The possible fragmentation patterns and ion nomenclature of flavonoid glycosides is illustrated on luteolin-7-*O*-rutinoside in Figure 4. For flavonoid glycosides, the classical nomenclature is proposed by Domon and Castello for glycoconjugates to denote major fragments: *^k,l^X_j_*, *Y_j_*, *Z_j_* represent the ions still containing the aglycone, where *j* is the number of interglycosidic bond broken (counted from the aglycon) and *k* and *l* denote the cleavage within the carbohydrate rings [34,35,36,37].

**Figure 5:**
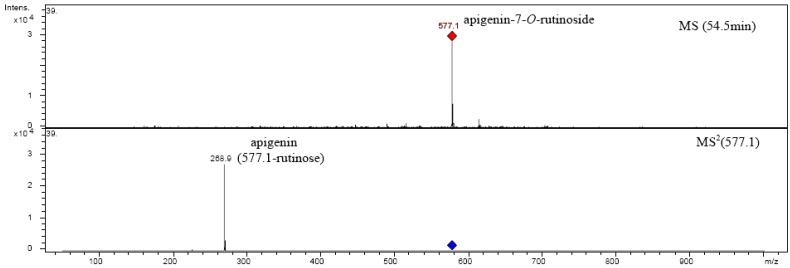
Fragmentation of apigenin-7-*O*-rutinoside.

**Figure 6:**
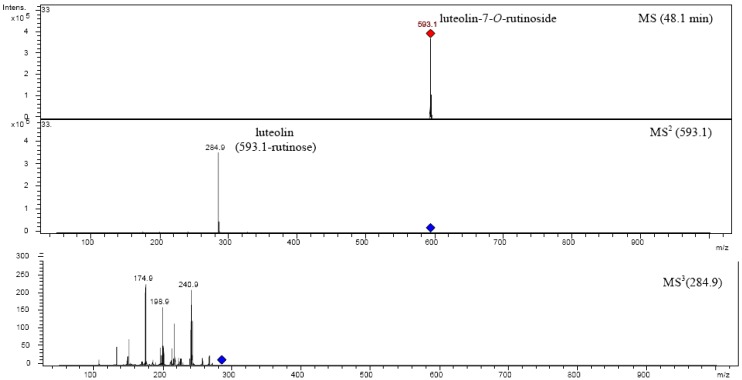
Fragmentation of luteolin-7-*O*-rutinoside.

In Figure 5 and Figure 6 are shown fragmentations of apigenin-7-*O*-rutinoside and luteolin-7-*O*-rutinoside identified in plant extract.

**Table 4:** Structures of identified flavonoids and results of screening and structure characterization.

Flavonoid	Mw	peak no.	*T_R_* (min)	UV_max_ (nm)	[M-H]^-^ *m/z*	HPLC-ESI-MS^n^ *m/z*
Apigenin	270.24	30	77.633	336	268.9	**MS^2^[268.9]**: 224.9, 250.9, 240.9, 204.9, 196.8, 180.9, 200.9, 148.8, 170.9, 116.9, 107.0
Luteolin	286.24	29	73.667	348	284.9	**MS^2^[284.9]**: 240.9, 266.9, 256.9, 242.9, 216.9, 198.9,174.8, 150.8,132.9
						**MS^3^[240.9]**: 196.9, 200.9, 212.9, 223.9, 148.9, 184.8, 170.8
Chrysoeriol	300.27	31	78.206	344	298.9	**MS^2^[298.9]**: 283.9
						**MS^3^[283.9]**: 255.9, 229.9, 226.7, 150.8
Apigenin-7-*O*-glucoside	432.38	26	58.205	334	431.0	**MS^2^[431.0]**: 268.9
						**MS^3^[268.9]**: 224.9, 250.9, 240.9, 204.9, 196.8, 180.9, 200.9, 148.8, 170.9, 116.9, 107.0
Apigenin-7-*O*-rutinoside	578.52	22	54.407	334	577.1	**MS^2^[577.1]**: 268.9
						**MS^3^[268.9]**: 224.9, 250.9, 240.9, 204.9, 196.8, 180.9, 200.9, 148.8, 170.9, 116.9, 107.0
Luteolin-7-*O*-glucoside	448.38	18	49.960	348	447.0	**MS^2^[447.0]**: 284.9
						**MS^3^[284.9]**: 240.9, 266.9, 256.9, 242.9, 216.9, 198.9, 174.8, 150.8, 132.9
Luteolin-7-*O*-rutinoside	594.52	16	48.022	348	593.1	**MS^2^[593.1]**: 284.9
						**MS^3^[284.9]**: 240.9, 266.9, 256.9, 242.9, 216.9, 198.9, 174.8, 150.8, 132.9
Chrysoeriol-7-*O*-rutinoside	608.55	27	59.324	344	607.1	**MS^2^[607.1]**: 298.9, 283.9
						**MS^3^[298.9]**: 283.9
Quercetin-3-*O*-galactoside	464.38	17	49.509	256	463.0	**MS^2^[463.3]**: 300.9
						**MS^2^[300.9]**: 178.8, 150.9, 120.8, 107.0, 168.7, 174.9 ,272.9, 256.8, 228.9
Quercetin-3-*O*-rhamnoside	448.38	24	56.407	256	447.0	**MS^2^[463.3]**: 300.9
				352		**MS^2^[300.9]**: 178.8, 150.8, 106.9, 120.8, 272.9, 228.9, 256.8
Quercetin-hexoside	464.39	15	47.301	356	463.0	**MS^2^[463.3]**: 300.9, 178.8
				256		**MS^2^[300.9]**: 178.8, 150.8, 106.8, 120.8, 272.9, 228.9, 256.9, 239.0, 162.9, 168.8
Quercetin-uronic acid	478.39	14	44.171	256	477.0	**MS^2^[477.0]**: 300.9, 178.8, 150.8
				355		**MS^2^[300.9]**: 178.8, 150.8, 106.9, 272.9, 228.9, 256.9, 192.8, 168.8
Chrysoeriol-uronic acid	476.42	28	68.380	266	475.0	**MS^2^[475.0]**: 298.9, 283.9, 254.8, 322.7, 390.7, 414.9
				354		**MS^2^[298.9]**: 283.9

### 2.5. Structure characterization of phenolic acids by MS^n^

**Figure 7:**
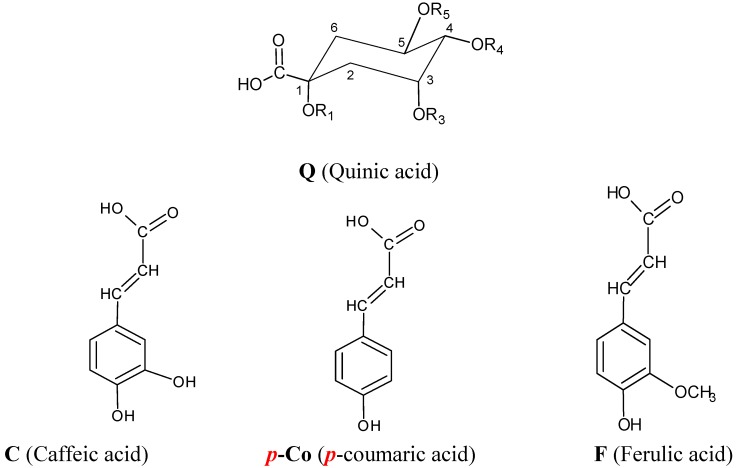
Structures of chlorogenic acids and associated cinnamic acids.

Structures of chlorogenic acids (CGA) are shown in Figure 7. CGA are widely distributed in plants, but few commercial standards are available, and precise identification of individual CGA in complex mixtures is therefore difficult. It is however possible to discriminate between each of the isomers on the basis of their fragmentation patterns and chromatographic resolution on a reversed phase packing. They were identified according to a hierarchical scheme for characterizing chlorogenic acids that has been developed by Clifford *et al*. [38,39,40]. The results of structural characterization and identification of phenolic acids are summarized in Table 5.

MS^n^ (n up to 3) identification data for phenolic acid derivatives are listed in Table 5. All monoacyl CGA give the parent ion [monoacyl CGA-H^+^]^-^ which identifies the CGA subclass. The diacyl CGA behaved similarly, giving the equivalent parent ion [diacyl CGA-H^+^]^-^. In further fragmentation examined compounds lost either caffeic acid or ferulic acid, yielding a [diacyl CGA-cinnamoyl-H^+^]^-^ as the MS^2^ base peak. These ions are identical to the parent ion obtained from CGA. Ions produced from diacyl CGA at MS^(n+1)^ are identical to those produced from monoacyl CGA at MS^n^.

4-acyl CGA is easy to identify by its "dehydrated" MS^2^ base peak at *m/z*~172.8 supported by MS^3^ ions at *m/z*~92.9 and at *m/z*~110.8. On the contrary, 1-acyl CGA and 3-acyl CGA produce an MS^2^ base peak at *m/z*~190.9 and MS^3^ ions at *m/z*~84.9, ~126.8, and ~172.9. The 3-CQA, which is commercially available, can be distinguished from 1-CQA, by its chromatographic resolution on reversed phase packing. 3-*p*CoQA is characterized by cinnamic acid-derived MS^2^ base peaks at *m/z*~162.9, 4-*p*CoQA by MS^2^ base peak at *m/z*~172.9 and 5-*p*CoQA as well as 5-FQA by MS^2^ base peaks at *m/z*~190.9. It is possible to distinguish monoacyl CGA on the basis of MS^1^ and MS^2^ spectra. MS^3^ spectra provide confirmation of these assignments. 

**Table:** 

Name	Abbreviation	R_1_	R_3_	R_4_	R_5_
1-*O*-caffeoylquinic acid	1-CQA	C	H	H	H
3-*O*-caffeoylquinic acid	3-CQA	H	C	H	H
5-*O*-caffeoylquinic acid	5-CQA	H	H	H	C
4-*O*-caffeoylquinic acid	4-CQA	H	H	C	H
3-*p*-*O*-coumaroylquinic acid	3-*p*CoQA	H	*p*-Co	H	H
5-*p*-*O*-coumaroylquinic acid	5-*p*CoQA	H	H	H	*p*-Co
4-*p*-*O*-coumaroylquinic acid	4-*p*CoQA	H	H	*p*-Co	H
3-*O*-feruloylquinic acid	3-FQA	H	F	H	H
5-*O*-feruloylquinic acid	5-FQA	H	H	F	H
4-*O*-feruloylquinic acid	4-FQA	H	H	H	F
1,3-di-*O*-caffeoylquinic acid	1,3-diCQA	C	C	H	H
1,4-di-*O*-caffeoylquinic acid	1,4-diCQA	C	H	C	H
1,5-di-*O*-caffeoylquinic acid	1,5-diCQA	C	H	H	C
3,4-di-*O*-caffeoylquinic acid	3,4-diCQA	H	C	C	H
3,5-di-*O*-caffeoylquinic acid	3,5-diCQA	H	C	H	C
4,5-di-*O*-caffeoylquinic acid	3,4-diCQA	H	H	C	C

**Table 5:** Identified phenolic acids and their esters (according to [38,39]).

**Compound**	**Mw**	**peak no.**	***T_R_*** (min)	**UV_max_** (nm)	**MS^1^ parent ion *m/z***	**MS^2^ base peak *m/z***	**MS^2^ secondary peaks *m/z*(intensity)**	**MS^3^ base peak *m/z***	**MS^3^ secondary peaks *m/z* (intensity)**
Caffeic acid	180.16	9	24.421	322	178.8	134.9	134.9 (3)	-	-
*p*-Coumaric acid	164.16	12	36.104	310	162.7	118.9	127.8 (20)	-	-
*o*-Coumaric acid	164.16	21	53.134	275	162.7	118.9	-	-	-
1-CQA	354.31	2	10.166	325	352.9	190.8	179.8 (40), 134.9 (11)	126.8	84.9 (75), 172.8 (70). 92.9 (60), 110.8 (20), 108.9 (25)
(1-*O*-caffeoylquinic acid)									
3-CQA	354.31	4	15.845	325	352.9	190.9	178.8 (20)	126.8	84.9 (70), 172.9 (50), 110.8 (30), 92.9 (65), 108.8 (35)
(3-*O*-caffeoylquinic acid)									
4-CQA	354.31	5	17.860	325	353.0	172.8	178.8 (75), 190.9 (20), 134.9 (15)	92.9	110.8 (45), 126,7 (2), 136.8 (17), 154.8 (25)
(4-*O*-caffeoylquinic acid)									
1,3-diCQA	516.47	19	50.806	325	515.0	352.9	190.9 (20), 179 (10), 334.9 (5)	190.8	179.8 (55), 134.9 (10)
(1,3-di-*O*-caffeoyquinic acid									
3,4-diCQA	516.47	23	55.859	325	515.0	352.9	172.9 (20), 178.8.1 (13), 190.8 (10), 202.9 (10), 254.9 (7), 298.9 (6), 334.9 (9)	172.8	178.9 (50), 190.7 (13), 134.8 (10)
(3,4-di-*O*-caffeoyquinic acid)									
4,5-diCQA	516.47	25	57.306	325	515.0	353.0	172.9 (19), 202.8 (8), 178.8 (10), 190.8 (4), 254.9 (7), 298.9 (8), 316.9 (4)	172.9	178.9 (40), 190.8 (20), 134.8 (7)
(4,5-di-*O*-caffeoyquinic acid									
3,5-diCQA	516.47	20	52.115	325	515,0	352.9	190,8 (10), 178.8 (4)	190.8	178.8 (50), 134.9 (10), 172.8 (4)
(3,5-di-*O*-caffeoyquinic acid									
QQCA (quinic-quinic-caffeic acid ester)	528.48	13	42.218	328	527.0	364.9	202.8 (25), 184.8 (7), 178.9 (3)	202.8	184.8 (25), 178.8 (13), 134.9 (7) ,140.9 (2)
5-FQA	368.34	11	28.154	322	367.0	190.9	172.9 (30), 160.9 (3), 136.9 (9)	84.9	172.9 (90), 126.6 (95)
(5-*O*-feruloylquinic acid)									
5-*p*CoQA	338.31	8	23.450	316	336.9	190.9	162.9 (4), 172.9 (2)	126.9	170.8 (90), 84.9 (60), 93.0 (40), 108.9 (4), 98.9 (10), 144.7(10)
(5-*p*-coumaroyl-quinic acid)									
4-*p*CoQA	338.31	10	24.933	315	336.9	172.9	162.8 (7), 190.9 (30), 118.9 (1), 154.9 (2), 110.9 (2)	-	-
(4-*p*-coumaroyl-quinic acid)									
3-*p*CoQA	338.31	3	14.854	310	337.0	162.9	190.9 (8), 172.9 (4), 118.9 (8)	118.9	-
(3-*p*-coumaroyl-quinic acid)									
GCA (gallic-caffeic acid ester)	332.28	1	6.215	315	330.9	168.8	192.9 (30), 210.8 (20), 270.9 (35), 150.7 (10), 124.8 (139	-	-
diCA (dicaffeic acid)	342.32	7	19.831	328	341.0	178.8	160.9 (35); 134.9 (15), 202.8(10), 280.9 (5), 250.9 (3)	-	-

**Figure 8:**
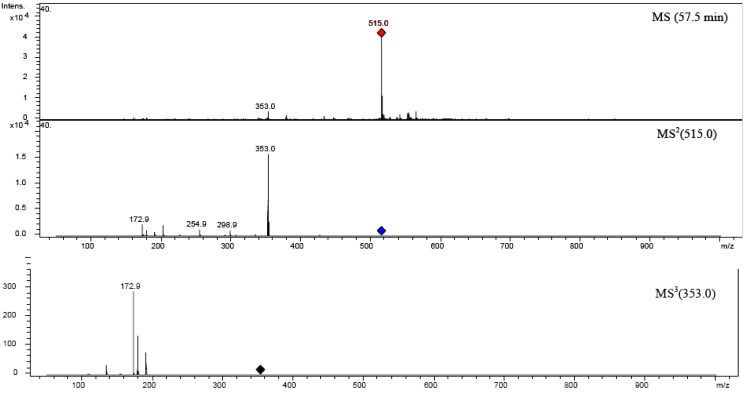
Fragmentation of 4,5-*O*-dicaffeoylquinic acid.

Methanolic extract of *C. platycarpos* gave 1,3-diCQA, 3,4-diCQA 3,5-diCQA and 4,5-diCQA chromatographic peaks, which were located by their parent ion at *m/z* 515 and distinguished by their fragmentation patterns and reversed phase chromatographic behaviors. Those CGA with greater number of free equatorial hydroxyl groups in the quinic acid residue are more hydrophilic than those with a greater number of free axial groups. The *vic* diCQA (3,4-diCQA and 4,5-diCQA) gave as the MS^3^ base peak at *m/z*~172.8, as was observed for 4-CQA and 4-*p*CoQA. This ion is characteristic for isomers with substitution at position 4 of quinic acid. The MS^2^ base peak for the *vic* diCQA is [4-CQA-H+]^-^, so 3,4-diCQA loses the caffeoyl moiety at position 3, and 4,5-diCQA initially must lose the substituent at position 5. Ion at *m/z*~172.8 was not detectable in the spectra of 3,5-diCQA and 1,3-diCQA, which give MS^3^ base peak at *m/z*~190.8. The two *vic* diCQA isomers are distinguished by the intensity of the MS^2^ "dehydrated" ion [CQA-H_2_O-H^+^]^-^ at *m/z*~334.9. In 3,4-diCQA it is more intense and in 4,5-diCQA it is barely detectable. Fragmentation of 4,5-*O*-dicaffeoylquinic acid is shown in Figure 8. 1,3-diCQA and 3,5-diCQA are distinguished by their fragmentation; *m/z* at 334.9 is not detectable in 3,5-diCQA, and by chromatographic behavior; 1,3-diCQA is more polar [38,39].

### 2.6. Chrysoeriol 7-O-α-L-rhamnosyl (1→6)-β-D-glucoside structure charcterization

**Figure 9:**
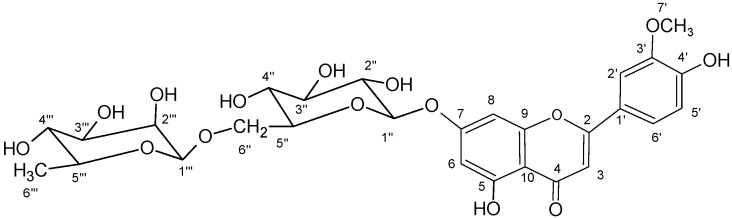
The structure of isolated chrysoeriol-7-*O-α*-l-rhamnosyl (1→6)-*β*-d-glucoside.

Chrysoeriol-7-*O*-*α*-l-rhamnosyl-(1→6)-*β*-d-glucoside was identified in *C. platycarpos* and isolated from the plant material by double preparative TLC for the first time. The structure of the isolated compound with atom numbering is shown in Figure 9.

**Figure 10:**
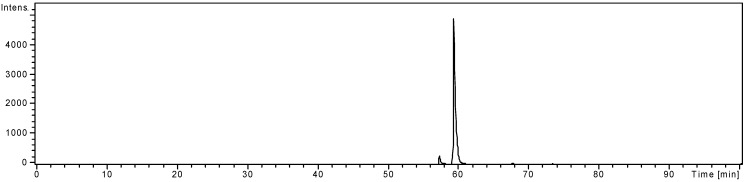
Total ion chromatogram of isolated chrysoeriol-7-*O-α*-l-rhamnosyl (1→6)-*β*-d-glucoside.

The purity of the isolated compound was checked by HPLC-DAD-MS and its total ion chromatogram is shown in Figure 10.

**Figure 11:**
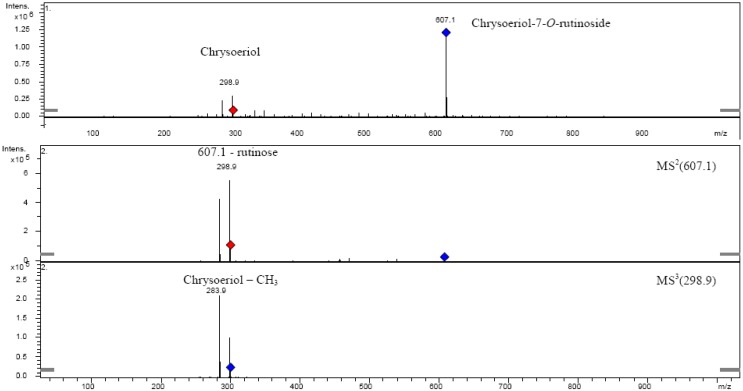
Fragmentation of chrysoeriol-7-*O-α*-l-rhamnosyl (1→6)-*β*-d-glucoside.

The fragmentation pattern of the isolated compound is shown in Figure 11, showing that the sugar containing hexose and methylpentose was attached on the same carbon, probably C-7. Because of lack of literature data for UV-VIS and MS^n^ for this compound, to prove the position of the function groups in the structure of the isolated compound it was further elucidated by UV with shift reagents, IR, ^1^H-NMR and ^13^C-NMR spectroscopic methods [41,42,43,44,45].

## 3. Experimental

### 3.1. General

Apigenin-7-*O*-glucoside, quercetin-3-*O*-galactoside, quercetin-3-*O*-rhamnoside, luteolin and apigenin were purchased from Fluka (Buchs, Switzerland). Luteolin-7-*O*-glucoside was from Chromadex (Irvine, USA). Chlorogenic acid (3-*O*-caffeoylquinic acid), caffeic acid, ferulic acid, isoferulic acid, *o*-coumaric acid, *p*-coumaric acid were from Acros Organics (Geel, Belgium). Chrysoeriol was from Extrasynthése (Genay, France). Chrysoeriol-7-*O*-rutinoside was isolated from the plant material, its purity was checked by HPLC and structure elucidated by ^1^H-NMR, IR, UV/VIS (with shift reagents) spectroscopic analysis and MS^n^ spectral data. Acetonitrile and methanol were HPLC grade from Merck (Darmstadt, Germany). Ethyl-acetate, chloroform, dichlormethane, acetic and formic acid of analytical grade were purchased from Kemika (Zagreb, Croatia). Water (0.055 µS/cm) was purified by a Milli-Q system from Millipore (Milford, USA). Ammonium formate was of mass spectrometry grade from Fluka (Buchs, Switzerland). PLC plates (20x20 cm glass plates, Silica gel 60, 1 mm thin layer) were purchased from Merck (Darmstadt, Germany). 

Analyses were performed on Agilent 1100 chromatograph equipped with a diode array detector and mass detector in series (Agilent Technologies, Waldbronn, Germany). A ZORBAX SB-C18, 4.6x250 mm, particle size 5 µm with suitable quard column was employed for the separation. The binary mobile phase consisted of solvents A (10 mM ammonium formate in water-acetonitrile 95:5 (v/v), pH 4.0) and B (10 mM ammonium formate in water-acetonitrile 50:50 (v/v), pH 4.0). The gradient elution started with 0% B and changed to 40% B in 65 minutes, then reached 100% B in 35 minutes. After each run the chromatographic system was set to 0% B in 10 minutes and equilibrated for 10 minutes. The flow rate was 1.0 mL/min and split out 200 μL/min to MS. Injection volume was 5µL.

Spectral data for all peaks were recorded in the range of 200-600 nm. The mass detector was an ion trap spectrometer (AgilentLC/MDS Trap VL) equipped with an electrospray ionization interface and controlled by LCMSD software. The ionization conditions were adjusted at 325 °C and 3.5 kV for capillary temperature and voltage, respectively. The nebulizer pressure was 35 psi and the nitrogen flow rate was 8 L/min. Collision-induced fragmentation experiments were performed in the ion trap using helium as a collision gas, with voltage cycles from 0.3 up to 2 V. All mass spectrometry data were recorded in negative ion mode. The screening was performed in full scan covering the range from m/z 50 up to 1000; multiple reaction monitoring (MRM) mode with ten ions set to be detected in one experiment, auto MS^n^ and manual MS^n^ (n up to 3) modes to fragment the major ions obtained in every step.

NMR spectra were recorded on a Bruker Avance 300 spectrometer, operating at 300 MHz for ^1^H- NMR and 75 MHz for ^13^C-NMR, using DMSO-d_6_ as solvent. Chemical shifts are expressed in δ (ppm) downfield from TMS as an internal standard, and coupling constants are reported in Hz. The IR spectra were recorded on a ATI Mattson FTIR spectrometer. UV spectra were recorded on Agilent UV-VIS 8453 diode array spectrophotometer.

### 3.2. Plant material

The aboveground parts of the plant *C. platycarpos*, were collected in the surroundings of Imotski, Croatia, in June 2008, identified by Professor Nikola Kujundžić, one of the authors, and deposited in the Department of Analytical Chemistry, Faculty of Pharmacy and Biochemistry, University of Zagreb, Croatia. The plant material was air dried, smashed into powder and stored in a dessicator.

### 3.3. Preparation of standard solutions

Standard stock solutions of six phenolic acids, four flavonoid glycosides and three flavonoid aglycones were made in methanol at a concentration of 1.0 mg/mL and stored in a refrigerator at -20 °C until use. All standard solutions were filtered through 0.45 μm filters and diluted as necessary with methanol.

### 3.4. Extraction

Air dried and grounded, aboveground parts of *C. platycarpos* were extracted as follows; about 1 g of accurately weight herb material was extracted with 20 mL of methanol at 60 °C, on magnetic stirrer (3000 rpm) for 30 min, three times. Filtrated extracts were combined, evaporated to dryness and dispersed in methanol-water (1:1, v/v). Chlorophyll was removed by extraction with chloroform. Purified extract was evaporated under reduced pressure to dryness, dispersed in 15.00 mL methanol, passed through 0.45 µm filter and stored at 4°C.

### 3.5. Isolation of chysoeriol-7-O-rutinoside by preparative liquid chromatography

Air dried plant material (500 g) was extracted with 1500 ml of methanol at 60 °C. Filtrated extract was evaporated to dryness and dissolved in 200 ml of methanol-water (1:1, v/v). Chlorophyll was removed by extraction with chloroform. Purified extract was evaporated under reduced pressure until methanol is removed. The water layer was partitioned against ethyl acetate. The ethyl acetate extract was again partitioned against water. This water extract was evaporated to dryness, dissolved in methanol and applied on silica gel 60, 20 x 20 cm, 1.0 mm thin layer TLC plates as a 15 cm band. The plate was developed using 100:25:10:10:11 (v/v/v/v/v) ethyl acetate-dichloromethane-formic acid-acetic acid-water The chromatographic band (*R*_F_=0.13) expected to contain the compound was scraped off and extracted with methanol. To purify the compound, preparative liquid chromatography was done once more using ethyl acetate-methanol-formic acid-water (100:13.5:2.5:10, v/v/v/v) as mobile phase and the same stationary phase. Purified compound (*R*_F_=0.25) was again extracted with methanol, filtered through 0.45 µm filter, evaporated to dryness, redissolved in methanol and checked for purity by HPLC-DAD-MS. UV λ_MeOH_ max (nm) 267, 272 sh, 342; + NaOMe 270, 277 sh, 391; +AlCl_3_ 273, 297 sh, 347, 394; +AlCl_3_ - HCl 275, 297 sh, 349, 390 nm; + NaOAc 269, 346; +NaOAc - H_3_BO_3_ 269, 350; IR *ν_max_* (KBr) 3400, 2958, 2831, 2790, 2716, 1600, 1412, 1360, 1148, 1115 cm^-1^; ^1^H-NMR: *δ* 1.97 (3H, d, *J_5''', 6'''_* = 8.6 Hz, H-6'''), 3.09 (2H, m, H-5'', H-5'''), 3.17 (2H, m, H-2'', H-3''), 3.21 (2H, m, H-4'', H-4'''), 3.32 (3H, s, H-7'), 3.88 (2H, m, H-2''', H-3'''), 3.90 (1H, m, H-6a''), 3.93 (1H, m, H-6b''), 4.26 (1H, d, *J_1'', 2''_* = 4.7 Hz, H-1'''), 4.85-5.11 (8H, m, OH -5, -4', -2'', -3'', -4'', -2''', -3''', -4'''), 6.79 (1H, s, H-6), 6.86 (1H, s, H-3), 7.03 (1H, s, H-8), 7.10 (1H, d, *J_5', 6'_* = 0.99, H-5'), 7.28 (1H, d, *J_5', 6'_* = 0.38 Hz, H-6'), 7.29 (1H, s, H-2'); ^13^C-NMR: *δ* 22.4 (C-6'''), 47.8 (C-7'), 69.4 (C-6''), 69.5 (C-5'''), 69.7 (C-4''), 69.8 (C-2'''), 69.9 (C-3'''), 70.0 (C-4'''), 70.1 (C-2''), 70.9 (C-5''), 71.0 (C-3''), 94.7 (C-8), 96.8 (C-1''), 98.0 (C-6), 101.2 (C-1'''), 105.6 (C-3), 106.1 (C-10), 109.6 (C-2'), 118.76 (C-5'), 121.0 (C-6'), 131.42 (C-1'), 144.7 (C-4'), 150.8 (C-3'), 157.8 (C-9), 161.9 (C-5), 164.6 (C-2), 167.6 (C-7), 181.3 (C-4).

## 4. Conclusions

Four phenolic acids (3-*O*-caffeoylquinic acid, caffeic acid, *p*-coumaric and *o*-coumaric acid), four flavonoid glycosides (luteolin-7-*O*-glucoside, apigenin-7-*O*-glucoside, quercetin-3-*O*-galactoside and quercetin-3-*O*-rhamnoside) and three flavonoid aglycones (luteolin, apigenin and chrysoeriol) were quantified in *C.* *platycarpos* extract. Chlorogenic acids (CGA) were found to be characteristic components of *C*. *Platycarpos*, among which caffeoylquinic (CQA), *p*-coumaroylquinic (*p*-CoQA), feruloylquinic (FQA) and dicaffeoylquinic (diCQA) acids have been identified. When standards were not commercially available, peaks were assigned primarily by means of their parent ion fragmentations supported by their UV spectrum and sequence of elution/retention time. The above mentioned flavonoid glycosides and phenolic acid esters, as well as isolated chrysoeriol-7-*O*-rutinoside, were identified as constituents of *C. platycarpos* for the first time.
